# Aquaporin-1 in peripheral nerve physiology and pathology: implications for uremic peripheral neuropathy

**DOI:** 10.1080/07853890.2026.2687216

**Published:** 2026-06-19

**Authors:** Xuan Li, Xingfu Ji, Jigang Shan, Xuexun Chen

**Affiliations:** ^a^ Department of Nephrology, Affiliated Hospital of Shandong Second Medical University, Weifang, China; ^b^ Affiliated Hospital of Shandong Second Medical University, Kidney Disease, School of Clinical Medicine, Shandong Second Medical University, Weifang, China; ^c^ The 960^th^ Hospital of the PLA Joint Logistics Support Force, Jinan, China

**Keywords:** Aquaporin-1, uremic peripheral neuropathy, peripheral nervous system, neuroinflammation, Schwann cells

## Abstract

**Background and objectives:**

Aquaporin-1 (AQP1) is a well-characterized transmembrane water channel widely expressed in the peripheral nervous system (PNS), where it contributes to water balance and regulation of the local neural microenvironment. Beyond its classical role in water transport, AQP1 has been implicated in edema formation, Schwann cell activity, nociceptive signaling, and inflammatory responses. However, these findings derive largely from experimental settings and vary in consistency across different models. Direct evidence supporting a role for AQP1 in uremic peripheral neuropathy (UPN) is currently lacking. This review re-examines the relevance of AQP1 in the PNS by integrating structural, cellular, and functional evidence, and evaluating how far current observations can be extended to the UPN context.

**Methods:**

We synthesized available literature on AQP1 in peripheral nerve biology, with particular attention to non-uremic models including nerve injury, hypoxia, osmotic stress, and inflammation. The applicability of these mechanistic clues to the complex metabolic, toxic, and inflammatory environment of uremia was critically assessed.

**Discussion:**

Alterations in AQP1 expression or function may plausibly contribute to peripheral nerve dysfunction under uremic conditions, potentially involving disrupted water homeostasis, Schwann cell instability, and altered neuroimmune interactions. However, these assumptions remain speculative. Existing models do not fully reproduce the uremic milieu, and no direct experimental validation in UPN is currently available.

**Conclusion:**

Current knowledge on AQP1 in UPN remains fragmented, necessitating a clearer distinction between established findings and hypothesis-driven interpretations. Further studies in uremia-specific animal models and clinical cohorts will be essential to clarify the pathophysiological and therapeutic significance of AQP1 in this condition.

## 1. Introduction

Uremic peripheral neuropathy (UPN) is a frequent complication of end-stage renal disease, particularly among patients receiving long-term dialysis, in whom reported prevalence exceeds 60% [1–3]. Clinically, UPN is associated with considerable impact on daily functioning and quality of life. Nevertheless, the mechanisms underlying its development remain unclear, and treatment options are largely limited to symptomatic management rather than targeted interventions [4–6].

Accumulating evidence suggests that disturbances in the neural microenvironment, which include osmotic imbalance, chronic inflammation, and metabolic toxicity, play important roles in UPN pathogenesis [7–11]. However, the molecular pathways by which these systemic abnormalities translate into localized peripheral nerve injury remain poorly defined.

Aquaporin-1 (AQP1), a highly selective water channel protein, is expressed in several components of the peripheral nervous system (PNS) and has been implicated in multiple neuropathological processes, including neural edema formation, inflammatory signaling, and modulation of neuronal excitability [12–14]. These findings suggest that AQP1 may be involved in regulating the neural microenvironment under pathological conditions.

Importantly, direct evidence linking AQP1 to UPN is currently lacking. Most available data are derived from related neuropathological conditions, such as diabetic peripheral neuropathy, nerve injury, and osmotic stress models. Therefore, its role in UPN should be interpreted with caution and regarded as hypothesis-generating rather than evidence-confirmed.

In this review, we integrate current knowledge on the structure, distribution, and functional roles of AQP1 in the PNS and propose a conceptual framework in which AQP1-mediated regulation of water homeostasis and neuroimmune interactions may contribute to peripheral nerve dysfunction under uremic conditions. By distinguishing established findings from indirect evidence, we seek to provide a balanced perspective and identify key directions for future research.

## 2. Overview of AQP1

### 2.1. AQP1 structure

As a membrane protein best known for its role in transcellular water movement, AQP1 enables water to rapidly cross biological membranes in response to osmotic gradients while maintaining high selectivity for water over other small solutes [15]. Structurally, AQP1 exists as a homotetramer. Rather than relying on a shared central channel, each monomer is composed of six transmembrane helices and forms its own water-conducting pore [16,17].

Notably, AQP1 permits water passage while largely excluding ions, including protons. This selective permeability is important for preserving osmotic equilibrium and cellular homeostasis [18]. Previous structural studies have shown that this feature depends mainly on two conserved elements, namely the asparagine–proline–alanine (NPA) motifs and the aromatic/arginine constriction region, both of which help define the narrow pathway through which water molecules pass.

Taken together, these structural characteristics explain the efficiency and selectivity of AQP1-mediated water transport. Under osmotic challenge, such properties may be especially relevant and may also have implications for pathological processes in the PNS, although direct evidence in this setting remains limited.

### 2.2. AQP1 distribution in the PNS

AQP1 is present in different cellular components of the PNS, including neurons and glial cells (Table 1). Early observations in sensory ganglia provided the first indication of its expression pattern. For example, Matsumoto et al. detected AQP1 mRNA in the trigeminal ganglion [12]. Subsequent work extended these findings to the dorsal root ganglion (DRG), where AQP1 is mainly observed in small- and medium-diameter neurons. In many cases, AQP1 is coexpressed with nociception-related markers such as substance P and transient receptor potential vanilloid (TRPV)1, consistent with its presence in C-fiber sensory neurons [19,20].

**Table 1.** AQP1 distribution in the peripheral nervous system.

Distribution Site	Cell Type	Species	Localization Characteristics
Dorsal Root Ganglion (DRG)	Small and medium-sized neurons (diameter <30μm)	Rat, Mouse	Co-localized with substance P and TRPV1 in C-fiber nociceptive neurons
	Satellite cells	Rat, Mouse	Surrounding neuronal cell bodies
	Schwann cells	Rat, Mouse	Expressed in both myelinated and unmyelinated nerve fibers
Trigeminal Ganglion	Neurons	Rat	First site where AQP1 mRNA expression was confirmed
	Satellite cells	Rat	Surrounding neuronal cell bodies
Sciatic Nerve	Schwann cells	Rat, Mouse	Enriched in paranodal regions at Schmidt-Lanterman incisures and nodes of Ranvier; co-localized with actin in paranodal regions
	Myelinated axons	Rat	Axolemma expression
	Unmyelinated axons (C-fibers)	Rat, Mouse	Expressed in Remak bundles
Enteric Nervous	GFAP-positive glial cells	Human	Myenteric plexus of esophagus and pancreas
	HuC/D-positive neurons	Rat	Myenteric plexus of ileum
Nodose Ganglion	Satellite cells	Rat	Surrounding neuronal cell bodies
Petrosal Ganglion	Satellite cells	Rat	Surrounding neuronal cell bodies
Periodontal Ruffini Endings	Axon terminals and terminal Schwann cells	Rat	Mechanoreceptor endings

Data are summarized from published studies and may vary across species and experimental conditions.

In peripheral nerves, AQP1 has also been identified in Schwann cells, including both myelinating and non-myelinating types. Its enrichment at Schmidt–Lanterman incisures and paranodal regions has been described in several studies, although the functional implications of this distribution are not entirely clear [21]. Apart from somatic sensory pathways, AQP1 expression has been reported in the enteric nervous system, where it is more commonly detected in glial fibrillary acid protein (GFAP)-positive glial cells, while expression in enteric neurons appears more restricted [13]. Additionally, AQP1 has been observed in satellite cells of cranial sensory ganglia and in certain mechanoreceptor endings, such as periodontal Ruffini endings.

Overall, these observations indicate that AQP1 is not confined to a single anatomical site within the PNS. Its presence across multiple cell types suggests potential involvement in local microenvironmental regulation, particularly processes related to water handling. At the same time, the extent to which this distribution translates into specific functional roles, especially under pathological conditions, remains to be clarified.

### 2.3. PNS functions of AQP1

#### 2.3.1. Water homeostasis and the myelin microenvironment

AQP1 expressed by Schwann cells is thought to contribute to water handling within the myelin microenvironment. In peripheral nerves, its distribution is not uniform. Previous studies have shown that AQP1 is enriched at Schmidt–Lanterman incisures and paranodal regions, where it is associated with cytoskeletal components such as actin [21]. This localization pattern is consistent with a role in water movement across the myelin sheath and preservation of local microenvironmental stability.

Evidence from cell-based experiments further supports a link between AQP1 and the water permeability of Schwann cells. Overexpression of AQP1 in cultured Schwann cells has been reported to increase membrane water permeability and induce morphological alterations. Under hypoxic conditions, its expression can also be upregulated through a hypoxia-inducible factor 1α (HIF-1α)-dependent mechanism [12]. These observations raise the possibility that AQP1 may be involved in processes related to nerve edema, particularly under stress conditions.

Even so, whether these findings can be directly extended to peripheral neuropathy remains unclear. Evidence in uremia-related settings is still limited.

#### 2.3.2. Neuronal excitability and pain modulation

Whether AQP1 contributes to nociception remains unsettled. Early studies in AQP1-deficient mice revealed reduced responses to thermal and chemical nociceptive stimuli, suggesting possible involvement in pain signaling [19]. Later electrophysiological and behavioral studies, however, failed to show obvious differences between knockout and wild-type animals [20].

At present, the available data do not support a consistent role for AQP1 in baseline pain perception. Any effect may be context-dependent. There are also reports linking AQP1 to neuronal excitability, possibly through interactions with sensory ion channels such as TRPV1 and TRPV4. Still, these observations come largely from non-uremic models, and their relevance to UPN remains uncertain [22].

#### 2.3.3. Nerve injury, inflammation, and regeneration

AQP1 expression appears to change dynamically after peripheral nerve injury. In sciatic nerve injury models, increased expression has been reported in the DRG and spinal cord during the early stage, followed by a later decline [23,24]. There is also evidence that AQP1 deficiency may impair axonal regeneration, suggesting that AQP1 could be involved in repair processes after nerve damage [25].

Most of evidence implicating AQP1 in neuroinflammation, however, are derived from central nervous system models, with comparatively limited data available in the PNS. For example, AQP1 deficiency has been associated with reductions in pro-inflammatory microglial polarization and cytokine release following traumatic brain injury [26]. Several signaling pathways, including nuclear factor κB (NF-κB), Janus kinase/signal transducer and activator of transcription (JAK/STAT), phosphoinositide 3-kinase/protein kinase B (PI3K/AKT), and extracellular signal-regulated kinase/mitogen-activated protein kinase (ERK/MAPK), have also been discussed in this context. Still, it remains unclear how far these findings apply to the PNS.

In peripheral nerves, AQP1 may influence endoneurial edema and the local inflammatory milieu through water transport effects on Schwann cells. Evidence for this possibility remains limited, especially in uremia-related settings.

## 3. Evidence linking AQP1 to peripheral neuropathy: Indirect clues relevant to UPN

Demyelination is a common feature of many peripheral neuropathies. In UPN, reported changes in nerve fibers include injury to both axons and myelin sheaths, with demyelination also observed in axons that appear relatively intact [21]. AQP1 is potentially involved in Schwann cell demyelination and repair processes, although direct evidence supporting this role in UPN is currently lacking. Segura-Anaya et al. [21] found that the localization of AQP1 at Schmidt–Lanterman incisures and paranodal regions corresponds to morphological changes commonly observed in demyelinating lesions, including paranodal retraction and Schmidt–Lanterman incisure collapse. In demyelinating diseases such as multiple sclerosis and Guillain–Barré syndrome, aquaporin-mediated water transport has been implicated in the regulation of tissue edema and microenvironmental homeostasis, although specific evidence regarding AQP1 remains limited [27,28]. Nevertheless, whether AQP1 expression changes in UPN-associated demyelination remains unknown. Based on its role in Schwann cell water transport and myelin homeostasis, it is plausible that AQP1 upregulation under hyperosmolar conditions relevant to uremia could contribute to Schwann cell edema and demyelination; however, this hypothesis requires experimental validation.

## 4. Pathophysiological features of UPN relevant to AQP1-mediated mechanisms

UPN presents with a variety of clinical features, including paresthesia, dyskinesia, and autonomic dysfunction. Patients often report pain, numbness, tingling, or burning sensations that usually start in the lower extremities and gradually spread upward [5]. Motor involvement may also occur, with weakness, muscle wasting, and diminished reflexes reported in affected patients [6,29]. Autonomic symptoms may include fluctuations in blood pressure, abnormal sweating, and gastrointestinal disturbances. Pathologically, UPN is mainly characterized by axonal degeneration, frequently accompanied by secondary segmental demyelination, although the mechanisms driving these changes remain incompletely understood. This clinicopathological profile provides a practical basis for considering candidate molecular processes relevant to UPN, including those potentially involving AQP1.

The pathophysiology of UPN is complex and likely reflects the combined effects of several interacting factors, including retention of uremic solutes, metabolic disturbance, electrolyte and acid–base imbalance, reduced neuroketolase activity, altered vascular reactivity, impaired transport across the blood–brain barrier, and chronic inflammation [7–11]. Among these contributors, uremic toxins are considered particularly important because of their neurotoxic potential. These retained solutes include guanidino compounds, advanced glycation end products, and tryptophan-derived metabolites, all of which have been implicated in neural injury. Their effects do not appear to arise from a single mechanism; rather, they may disrupt neuronal function at multiple levels and thereby contribute to cellular damage and neurological dysfunction.

Secondary hyperparathyroidism may further worsen neurological impairment in the uremic setting. Electrophysiological studies in patients with UPN have demonstrated slowing of nerve conduction velocity, a finding generally consistent with diffuse axonal neuropathy. Distal axonal degeneration is also thought to be related to metabolic dysfunction within neurons, at least in part because of direct exposure to retained uremic solutes. In addition, several circulating markers, including C-reactive protein (CRP), parathyroid hormone (PTH), serum albumin (Alb), and hemoglobin (Hb), have been reported to change in patients with UPN. Whether these alterations directly reflect disease severity remains uncertain, but they may still provide useful clinical context [30].

UPN is likely driven by multiple concurrent abnormalities rather than a single mechanism. In advanced chronic kidney disease, retention of uremic solutes, metabolic disturbance, electrolyte imbalance, and reduced enzymatic activity may collectively promote neural dysfunction and structural injury. Some of these changes may intersect with pathways relevant to AQP1, particularly those involved in water handling and neuroinflammatory responses. At present, however, direct evidence supporting this link in UPN remains limited.

## 5. A Conceptual framework linking AQP1 to UPN

**Figure 1.**
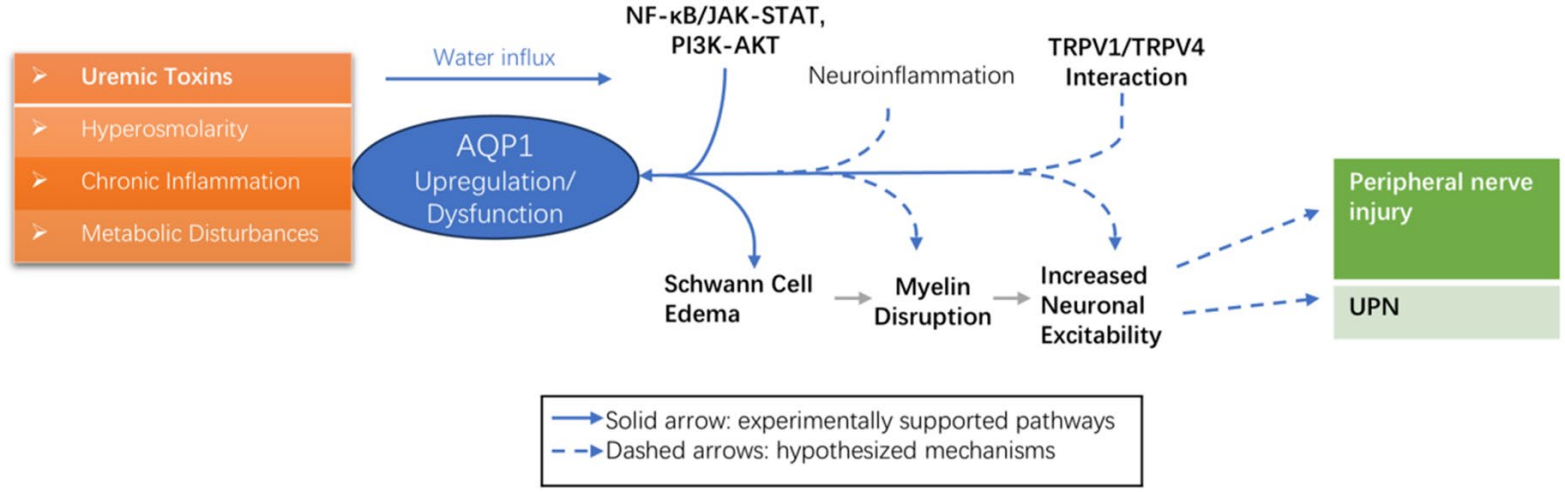
A conceptual framework for AQP1 in peripheral nerve dysfunction: implications for uremic neuropathy. Uremic conditions, including toxin accumulation, hyperosmolarity, and chronic inflammation, may induce dysregulation of AQP1 expression in the peripheral nervous system. Altered AQP1 function may contribute to Schwann cell edema and myelin instability through increased water influx, modulate neuroinflammatory responses *via* multiple signaling pathways, and influence neuronal excitability through interactions with ion channels. These interconnected mechanisms may collectively contribute to peripheral nerve injury and the development of UPN. Solid arrows indicate established mechanisms, while dashed arrows represent hypothesized pathways requiring further validation.

Despite a lack of direct evidence connecting AQP1 with UPN, findings from related neuropathological settings support AQP1 as a possible contributor to this condition. Rather than acting through a single mechanism, AQP1 may operate at the interface of osmotic stress, neuroinflammatory responses, and neuronal dysfunction, thereby providing one possible link between systemic uremic disturbances and peripheral nerve injury (Figure 1).

### 5.1. Osmotic stress and schwann cell edema

Uremia is accompanied by retention of metabolic solutes and changes in extracellular osmolarity, both of which may alter the microenvironment of peripheral nerves. AQP1 expression reportedly increases under hyperosmolar conditions, possibly as part of an adaptive response that facilitates transmembrane water flux [31,32]. However, most of these observations come from non-neural or non-mammalian systems, leaving their relevance to Schwann cells or peripheral nerves uncertain.

Because Schwann cells are essential for myelin maintenance, altered AQP1 expression in these cells could disturb local water balance and promote cellular swelling or endoneurial edema. Experimental studies have shown that AQP1 overexpression increases water permeability and is accompanied by morphological changes in Schwann cells, whereas reduced AQP1 expression may attenuate edema formation [12,33]. In UPN, where demyelination and Schwann cell dysfunction are common pathological findings, such changes could be relevant to myelin instability and impaired nerve conduction. Even so, this interpretation is based mainly on non-uremic models, thus requiring confirmation in uremia-specific settings.

### 5.2. Inflammatory signaling and neuroimmune modulation

Chronic low-grade inflammation is a well-recognized feature of uremia and is thought to contribute to peripheral nerve injury [7–11]. AQP1 has been linked to several signaling pathways involved in inflammatory regulation, including NF-κB, JAK/STAT, PI3K/AKT, and ERK/MAPK [26]. Even so, most of the available evidence comes from central nervous system models or non-uremic conditions, making its relevance to peripheral neuroinflammation uncertain.

Experimental studies have shown that loss of AQP1 in the central nervous system can reduce microglial activation and lower the production of pro-inflammatory cytokines such as tumor necrosis factor-α (TNF-α) and interleukin-6 (IL-6), changes that may lessen neuronal injury [26]. Whether similar effects occur in the PNS remains unclear. In peripheral nerves, AQP1 may instead influence the inflammatory milieu more indirectly, for example through its effects on Schwann cell function and local fluid balance. At present, the role of AQP1 in peripheral neuroinflammation should be regarded as provisional rather than established.

### 5.3. Neuronal excitability and neuropathic pain

Neuropathic pain is a common clinical feature of UPN and is closely related to abnormal excitability of sensory neurons. AQP1 has been detected in subsets of small sensory neurons that also express nociception-related markers such as TRPV1, which suggests a possible association with pain signaling [19,22].

Mechanistically, in addition to its role in water transport, AQP1 may affect local membrane microenvironments and thereby influence ion channel activity involved in sensory transduction. Findings from nerve injury models further support a possible link between AQP1 and pain sensitivity [22]. However, these data are derived mainly from non-uremic settings, and it remains uncertain whether the same mechanisms operate in UPN. For this reason, any proposed role of AQP1 in uremic neuropathic pain should be considered tentative.

### 5.4. Integrated pathophysiological model

The findings discussed above support a tentative model in which AQP1 links several features of the uremic milieu to peripheral nerve injury. First, osmotic disturbance may alter extracellular osmolarity under uremic conditions, favoring increased AQP1 expression in Schwann cells leading to potential effects on water handling, cellular swelling, and myelin stability. Second, considering that AQP1 has been associated with signaling pathways relevant to neuroinflammatory regulation, chronic inflammation may represent another component. Third, AQP1 may influence neuronal excitability to contribute to pain-related manifestations.

Together, these pathways could help explain several abnormalities observed in UPN, including demyelination, axonal injury, and sensory dysfunction. However, this model remains provisional. Most of the supporting evidence is indirect, and direct confirmation in uremia-specific systems is still lacking.

### 5.5. Therapeutic implications

Given its reported links to water transport, inflammatory signaling, and neuronal function, AQP1 may represent a potential therapeutic target in peripheral neuropathy. Experimental studies have shown that reduced AQP1 expression can attenuate neuropathic pain in models of DRG compression [22]. Pharmacological agents such as tanshinone IIA have also been reported to modulate AQP1 expression and improve peripheral nerve function in experimental settings.

Whether these observations are relevant to UPN remains uncertain. Existing studies have largely been conducted in non-uremic models, so their applicability to the uremic setting cannot yet be assumed. Further work is needed to clarify how AQP1 expression changes in uremia and whether interventions directed at this pathway can modify disease-related nerve injury.

## 6. Summary and prospect

Current evidence does not establish a direct role for AQP1 in UPN, but does identify this channel as a plausible mediator linking uremic disturbance and peripheral nerve injury. The main lines of evidence from related models suggest that AQP1 may influence water balance, neuroinflammatory responses, Schwann cell dysfunction and sensory signaling. These processes overlap with key features of UPN, although the links remain largely inferential rather than proven.

Most of the supporting observations come from non-uremic models [12,21,26]. which limits direct extrapolation to UPN. Studies in uremia-specific systems are still needed to determine whether AQP1 expression changes in this disease setting, how it is regulated, and whether it contributes to nerve edema, myelin instability, inflammatory signaling, or pain-related dysfunction.

Targeting AQP1-mediated pathways may therefore represent a promising exploratory therapeutic strategy for UPN, but this possibility requires direct experimental and clinical validation.

## Data Availability

The authors confirm that no data were generated or analyzed in this study. This is a narrative review, and all information is derived from published literature cited in the references.
